# miR-126&126* Restored Expressions Play a Tumor Suppressor Role by Directly Regulating ADAM9 and MMP7 in Melanoma

**DOI:** 10.1371/journal.pone.0056824

**Published:** 2013-02-21

**Authors:** Nadia Felli, Federica Felicetti, Anna Maria Lustri, M. Cristina Errico, Lisabianca Bottero, Alessio Cannistraci, Alessandra De Feo, Marina Petrini, Francesca Pedini, Mauro Biffoni, Ester Alvino, Massimo Negrini, Manuela Ferracin, Gianfranco Mattia, Alessandra Carè

**Affiliations:** 1 Department of Hematology, Oncology and Molecular Medicine, Istituto Superiore Sanità, Rome, Italy; 2 Laboratory for the Technology of Advanced Therapies (LTTA), Department of Experimental and Diagnostic Medicine, University of Ferrara, Ferrara, Italy; 3 Institute of Translational Pharmacology, National Research Council, Rome, Italy; The Ohio State University, United States of America

## Abstract

The abnormal expression of several microRNAs has a causal role in tumorigenesis with either antineoplastic or oncogenic functions. Here we demonstrated that miR-126 and miR-126* play a tumor suppressor role in human melanoma through the direct or indirect repression of several key oncogenic molecules. The expression levels of miR-126&126* were elevated in normal melanocytes and primary melanoma cell lines, whereas they markedly declined in metastatic cells. Indeed, the restored expression of miR-126&126* in two advanced melanoma cell lines was accompanied by a significant reduction of proliferation, invasion and chemotaxis *in vitro* as well as of growth and dissemination *in vivo*. In accordance, the reverse functional effects were obtained by knocking down miR-126&126* by transfecting antisense LNA oligonucleotides in melanoma cells. Looking for the effectors of these antineoplastic functions, we identified ADAM9 and MMP7, two metalloproteases playing a pivotal role in melanoma progression, as direct targets of miR-126&126*. In addition, as ADAM9 and MMP7 share a role in the proteolytic cleavage of the HB-EGF precursor, we looked for the effectiveness of this regulatory pathway in melanoma, confirming the decrease of HB-EGF activation as a consequence of miR-126&126*-dependent downmodulation of ADAM9 and MMP7. Finally, gene profile analyses showed that miR-126&126* reexpression was sufficient to inactivate other key signaling pathways involved in the oncogenic transformation, as PI3K/AKT and MAPK, and to restore melanogenesis, as indicated by KIT/MITF/TYR induction. In view of this miR-126&126* wide-ranging action, we believe that the replacement of these microRNAs might be considered a promising therapeutic approach.

## Introduction

Malignant melanoma is the most aggressive form of skin cancer whose incidence has more than tripled in the white population during the last 20 years. Although surgical excision is mostly a definitive treatment at the early stages of the disease, at present standard treatments are ineffective after metastatic dissemination and patients with advanced disease have a severe prognosis [1].

Non-coding small RNAs, termed microRNAs, have been demonstrated to play functional roles in all the major cellular processes, including tumorigenesis, where they can act as oncogenes as well as tumor suppressor genes, providing a new level of molecular regulation [2].

We focused on miR-126&126* as they appeared strongly downmodulated in a panel of melanoma cell lines at different stages of progression compared with normal human melanocytes. MiR-126 and its complement miR-126*, are hosted by intron 7 of the epidermal growth factor-like domain 7 (EGFL7) gene, a secreted factor implicated in cellular responses such as cell migration and blood vessel formation [3]. Besides a role in blood vessel growth and inflammation [4], [5] a number of studies reported miR-126&126* expression and function in various types of cancer where these microRNAs act as tumor suppressors [6]–[8]. Interestingly miR-126 has been reported to suppress metastasis of breast cancer cells to different distant sites, at least in part by reducing endothelial cell recruitment [9]. At present no data exist on miR-126&126* possible role in melanoma.

Our results show miR-126&126* downregulation associated with tumor progression and the antineoplastic role deriving from their restored expression in metastatic melanomas *in vitro* as well as *in vivo*. Looking for downstream target genes, we demonstrate miR-126&126* capabilities to directly regulate a disintegrin and metalloprotease domain 9 (ADAM9) and metalloprotease 7 (MMP7), whose aberrant expressions have been associated with a wide variety of cancers. ADAM9 and MMP7 are involved in cell-cell and cell-matrix interaction, are able to cleave and release a number of molecules with important role in tumorigenesis, resulting causally involved in tumor formation and progression [10]–[14]. In melanoma ADAM9 protein expression is restricted to the cells within the invading front, being detectable in tumor cells and in peritumoral stromal fibroblasts, and absent in fibroblasts distal to the tumor site [15]. Expression studies in primary cutaneous and metastatic melanomas also revealed MMP7 association with tumor cell invasion and progression suggesting its prognostic value in predicting the clinical behavior of melanoma [16].

MiR-126&126* play their tumor suppression function through the direct inhibition of ADAM9 and MMP7, in turn impairing the activation of their common target heparin-binding EGF-like growth factor **(**HB-EGF) [12]–[14]. In addition, as shown by our gene profiling studies, miR-126&126* appear to converge into a wide-ranging action as, beside ADAM9 and MMP7, they regulate a number of key oncogenic pathways. In this view we discuss the option of miR-126&126* restored expression as a novel therapeutic approach for treating melanoma diseases.

## Results

### miR-126&126* Expression Level is Downmodulated during Melanoma Progression

**Figure 1:**
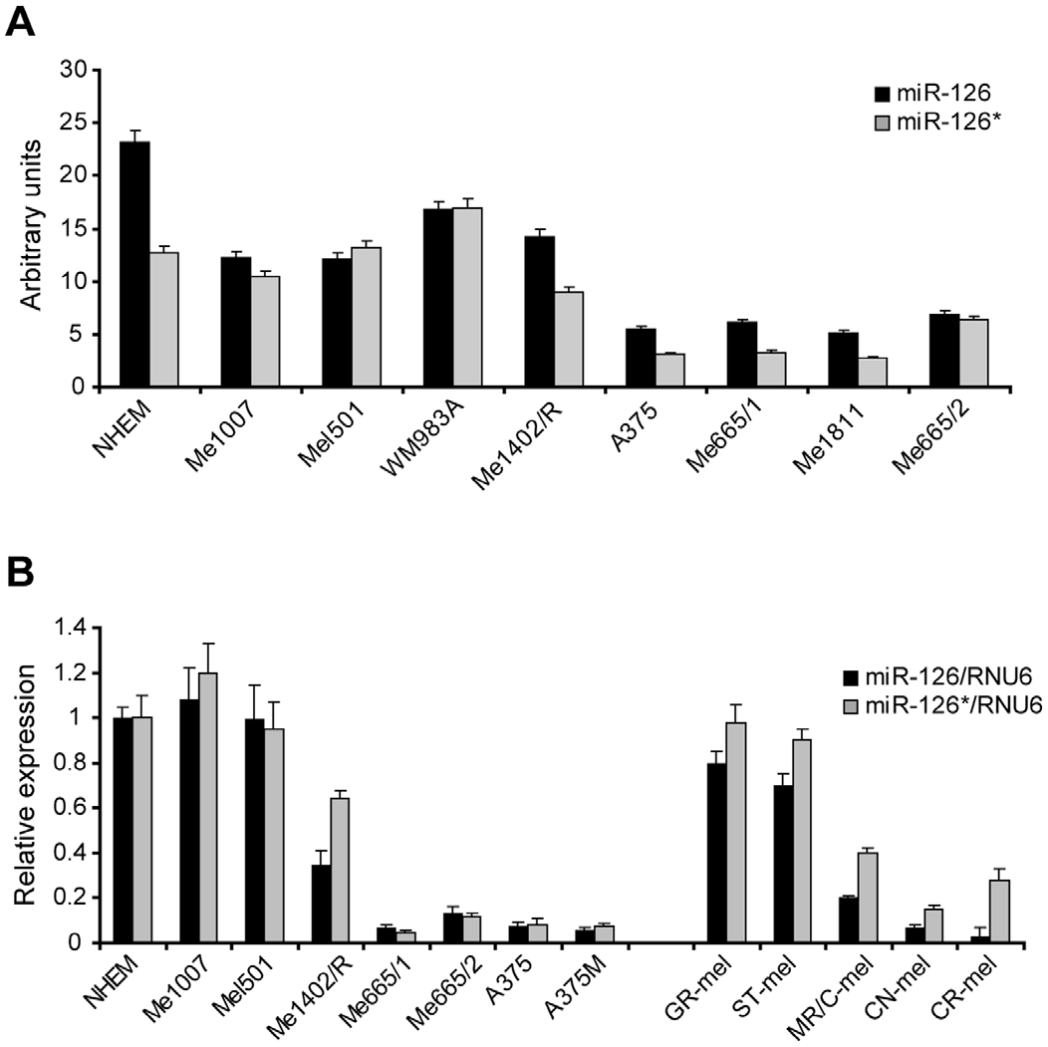
MiR-126 and miR-126* expression in normal human melanocytes and melanoma cell lines. A) Expression levels obtained by microRNA expression profiling; **B)** Real-time PCR in representative melanoma cell lines (left side) and in early passage cells from patients (right side) compared to normal human melanocytes (NHEM). Relative miRNA expression levels were normalized on RNU6B levels. Columns, mean±SD of at least two independent experiments.

Based on previous microRNA expression profiles (our unpublished results), we focused our attention on miR-126 and miR-126* levels in a panel of melanoma cell lines at different stages of progression, including primary vertical growth phase (VGP) melanomas, as well as subcutaneous and lymph-node metastases (Figure 1A) [17]. Quantitative real-time RT-PCR analysis (qRT-PCR) (Figure 1B left side) confirmed the microarray data (Figure 1A) showing a significant inverse correlation respect to melanoma malignancy. In particular, a significant drop of miR-126&126* levels was detectable in metastatic respect to primary melanomas (Figure 1A and B), suggesting a functional association of these microRNAs with melanoma advancement. To strengthen our results, ruling out any possible artifactual result due to *in vitro* cell culture, this expression pattern was confirmed in some representative early passage cells obtained from melanoma bioptic samples (Figure 1B right side). Note that the high level of miR-126&126* detected in normal human melanocytes (Figure 1A, B), sustain their physiological role.

### Evaluation of miR-126&126* Functional Effects on Melanoma Malignancy

To investigate the functional role of miR-126&126* on tumorigenesis, we stably transduced miR-126&126* in two advanced melanoma cell lines expressing low endogenous levels of these microRNAs. A genomic fragment encompassing miR-126&126* was cloned into either a constitutive or an inducible lentiviral system for transducing Me665/1 and A375M metastatic melanoma cell lines. qRT-PCR confirmed miR-126&126* overexpression in miR- versus empty vector-transduced melanoma cells (Figure S1). Specifically, we obtained ∼3-fold increase of miR-126 and miR-126* levels in the constitutively transduced Me665/1 melanoma (Tween-126&126* *vs* Tween), whereas a significantly higher expression (100-fold increase, mean value) was achieved by the inducible system in the A375M melanoma cell line (TripZ-126&126* *vs* TripZ). The requirement of an inducible vector derived from a possible miR-126&126*-dependent counter selection observed in the constitutively expressing cells.

**Figure 2:**
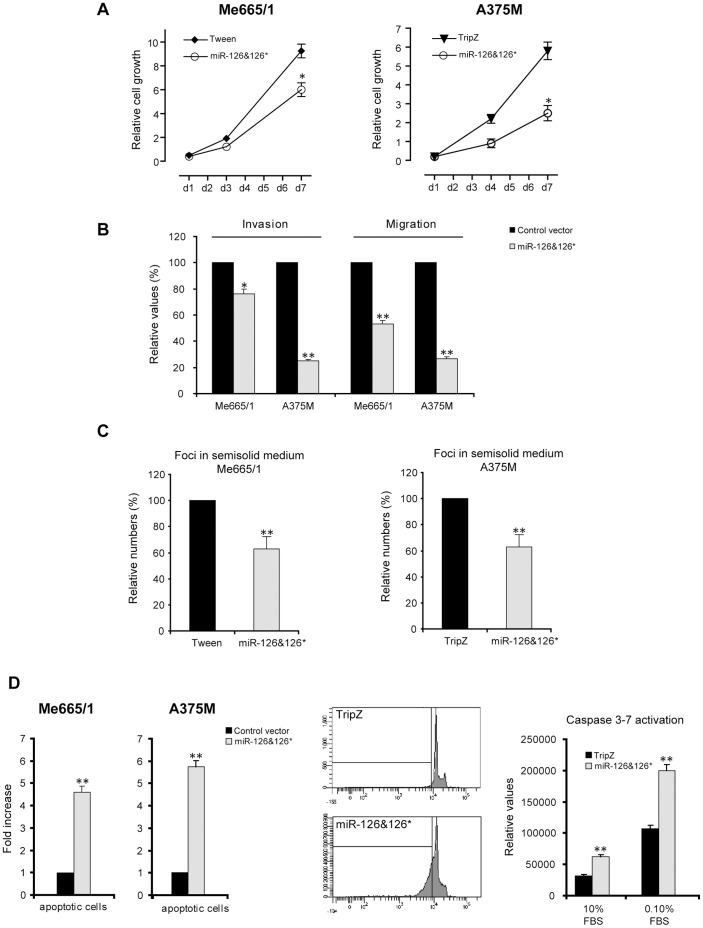
Overexpression of miR-126&126* in metastatic melanoma cell lines: *in vitro* functional studies. **A)** Cell proliferation, **B)** invasion (left), chemotaxis (right) and **C)** cell colony growth in semisolid medium assays. **D)** Relative quantitation of apoptotic cells in Me665/1 and A375M cell lines (left), evaluation of sub-G0 cells (middle) and relative levels of activated caspase 3 and caspase 7 in A375M cells (right). Columns, mean±SD of a minimum of two independent experiments; *P<0.05; **P<0.01.

Evaluating the involvement of miR-126&126* in melanoma cell growth, we observed a significant 30 to 50% decrease of proliferation in miR-126&126* overexpressing cells compared with controls (Figure 2A). As a next step we utilized some *in vitro* specific assays to verify whether miR-126&126* ectopic expression could influence the main biological properties governing melanoma dissemination. We assayed the invasive capabilities of these melanoma cells by using a Boyden chamber assay in miR-126&126*-transduced cells, observing decreased invasion and chemotaxis, ranging from 60–70% in A375M to 30–40% in Me665/1 (Figure 2B). It is interesting to note that, as already observed for proliferation, the inhibitory effects appear directly related to miR-126&126* over-expression levels. The outcomes deriving from miR-126&126* enforced expression were also evaluated on melanoma capability of forming foci in agar semisolid medium. Results showed a significant decrease (approximately 40%) of the number of foci in both cell lines (Figure 2C).

To further characterize the effects of miR-126&126* re-expression in A375M and Me665/1 cells, we evaluated their possible role in the apoptotic process. By using flow cytometer analyses, through propidium iodide incorporation and sub-G0 cell evaluation (Figure 2D, left and middle), a significant induction of apoptotic cells was observed in low serum condition. Looking for the underlying molecular bases, we focused on the activation levels of caspase 3 and 7, key molecules of the apoptotic cascade. In the A375M cells we observed a miR-126&126*-dependent induction of the active forms of caspase 3 and 7 respect to control cells, either in 10% or 0.1% serum (Figure 2D, right).

**Figure 3:**
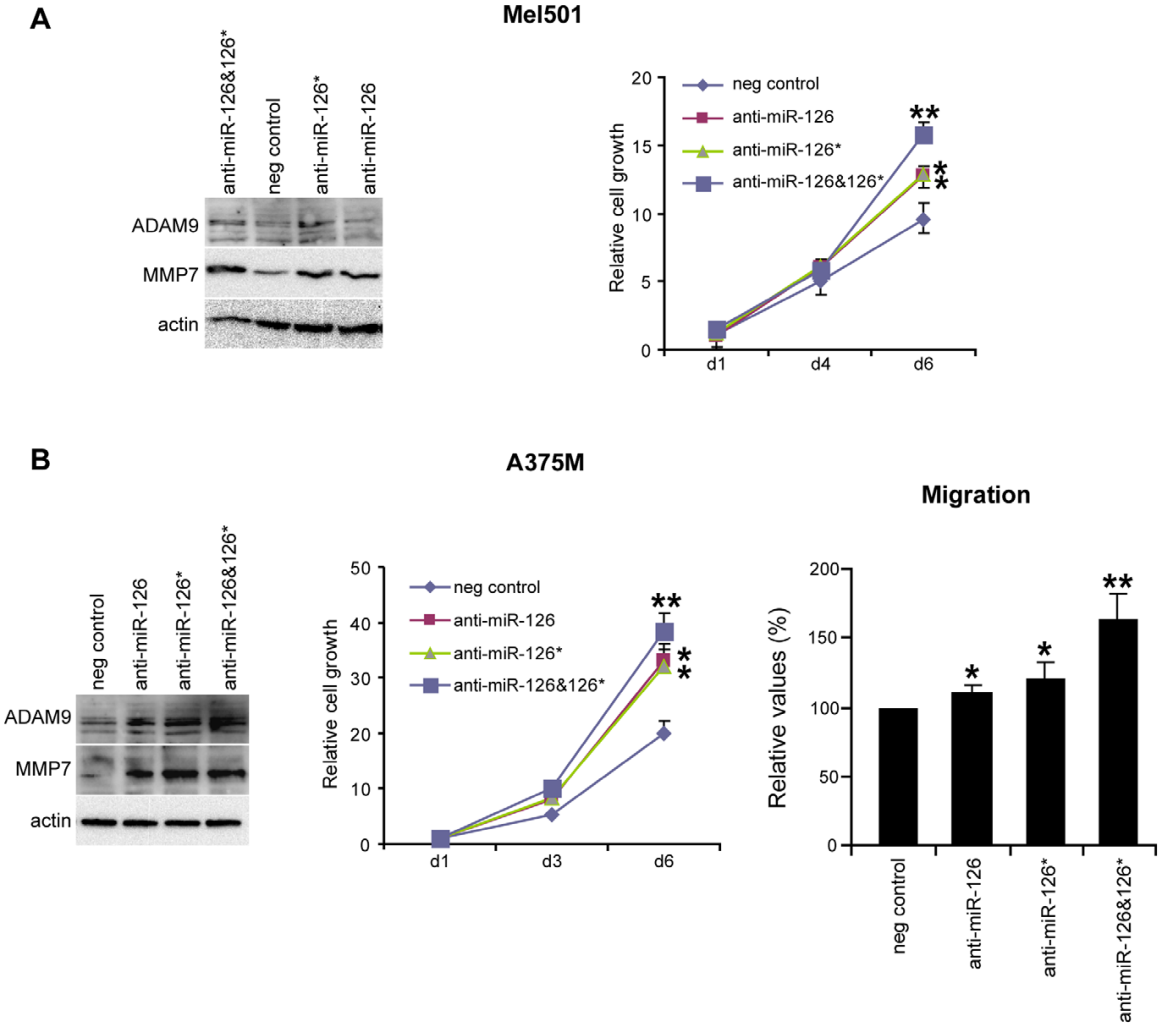
Effects of miR-126&126* knockdown evaluated in Mel501 primary and A375M metastatic melanoma cell lines. **A)** Representative WB analysis of ADAM9 and MMP7 target genes (left); cell growth proliferation analysis (right). **B)** WB analysis of ADAM9 and MMP7 (left), cell growth proliferation (middle) and migration (right). Actin was utilized as internal loading control.

The opposite functional effects were obtained when miR-126&126* were knocked out in melanoma cell lines by transfecting anti-miR-126 and/or 126* Locked Nucleic Acid (LNA) oligonucletides, either in the primary Mel501 or the metastatic A375M cell lines. As shown in Figure 3, in both cell lines the abrogation of miR-126&126* induced a more malignant phenotype associated with microRNA target derepression (Figure 3A, B). Specifically, miR-126&126* downregulation increased the rate of cell proliferation in both melanoma cell lines, and induced a significant increment of the chemotactic capabilities in the metastatic A375M cells. These effects were more evident when both miRs were silenced (Figure 3). It is important to evidence that the transient knock down of miR-126&126* in the primary Mel501 cell line, *per se* unable to disseminate, was not sufficient to induce its chemotactic capability (data not shown).

### *In vivo* Studies

**Figure 4:**
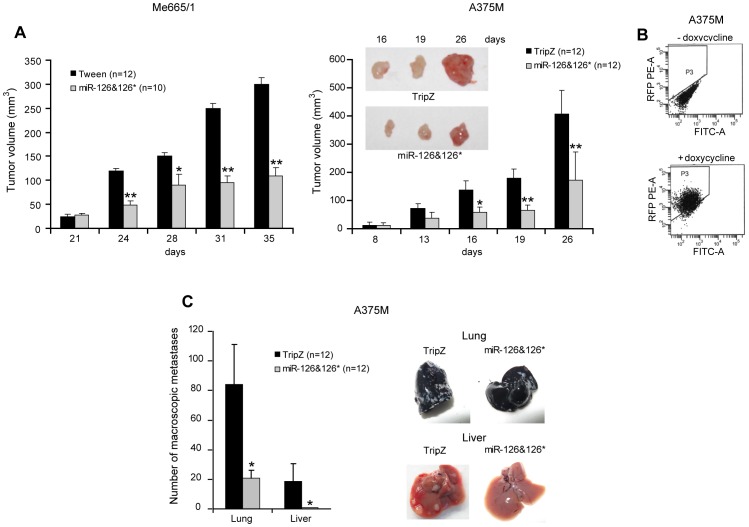
Effects of miR-126&126* overexpression in *in vivo* xenograft models. A) *In vivo* tumor growth at different time points deriving from vector- or miR-126&126*-infected metastatic melanoma cell lines s.c. injected into athymic nude mice. Representative nodules are shown as an insert. **B)** Representative flow cytometer results of doxycycline-dependent induction of the RFP reporter in cells deriving from dissociated tumor nodules. **C)** Total number (left) and representative pictures (right) of lung and liver macroscopic metastases obtained by intravenous injection of the A375M cell lines. Columns, mean±SD of two independent experiments; **, *P*<0.001; *, *P*<0.05.

We analyzed miR-126&126* potential role in *in vivo* xenograft models of melanoma. Control as well as miR-126&126*-infected metastatic cell lines (Me665/1 and A375M) were subcutaneously injected in natural killer (NK)-depleted athymic nude mice. Tumor growths were compared and followed over approximately 4 weeks. As shown (Figure 4A), the tumor volumes of miR-126&126*-expressing Me665/1 and A375M cells were significantly reduced when compared to their respective controls at all the analyzed time points. In addition, searching for differences in the number of macroscopic metastases, *in vivo* experiments were run with i.v. injected A375M cells, capable of distant dissemination. The effectiveness of doxycycline induction was monitored by detecting the red fluorescent reporter protein (RFP) in melanoma cells after tumor mass dissociation (Figure 4B). A significant miR-126&126*-dependent decrease of lung and liver metastases was observed. In particular, lung metastases count under microscope strongly dropped down (84±24 *vs* 21±4, mean values±SD), whereas dissemination to other abdominal organs (i.e liver) was essentially restricted to control cells (Figure 4C, left). Representative organs are shown (Figure 4C, right).

### Expression Analysis of miR-126&126* Putative Target Genes: ADAM9, MMP7 and OPN

**Figure 5:**
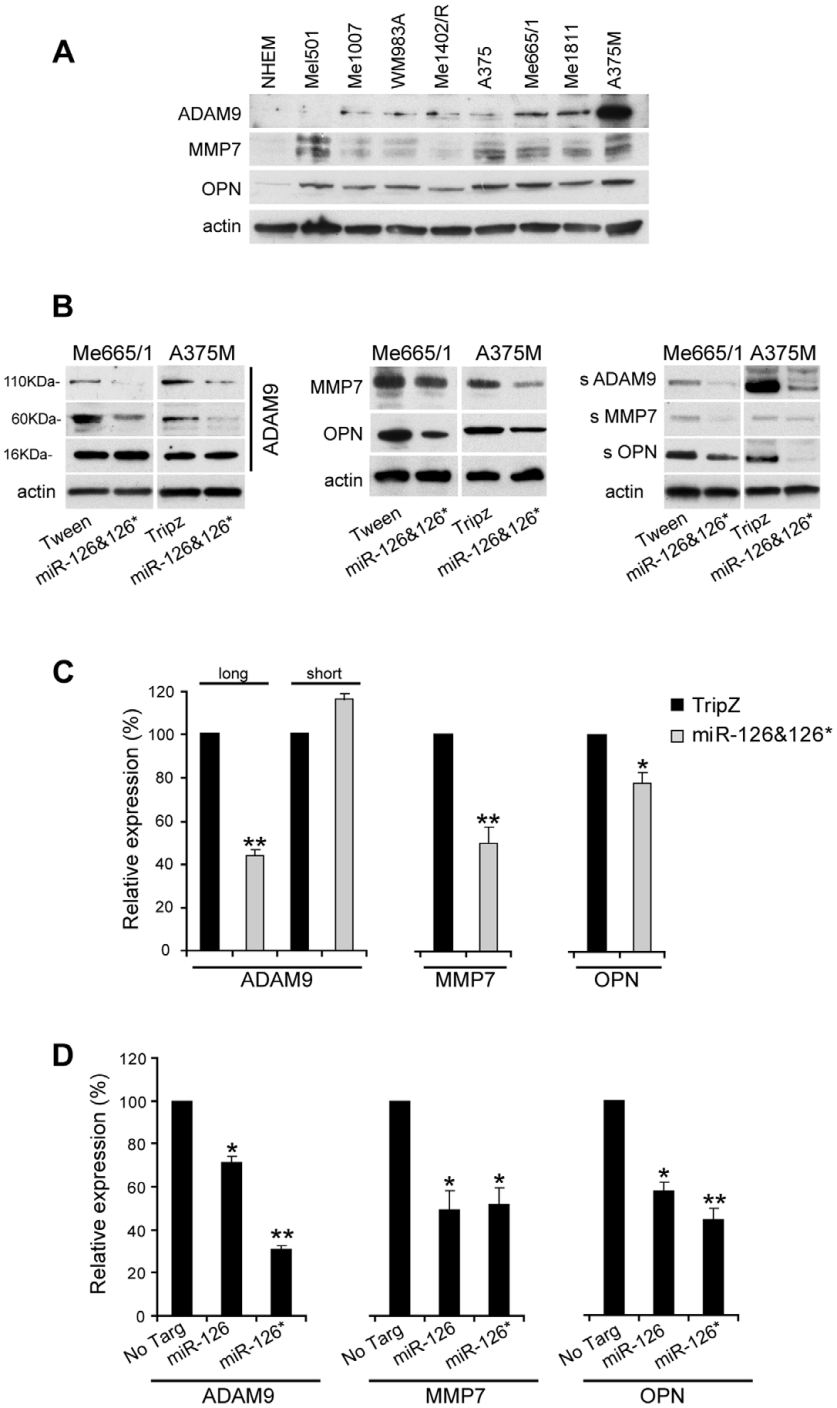
miR-126&126* target genes: ADAM9, MMP7 and OPN. Representative Western blot of **A)** ADAM9, MMP7 and OPN in normal human melanocytes (NHEM) and in a panel of melanoma cell lines, **B)** ADAM9 isoforms (left), MMP7 and OPN (middle) and corresponding secreted forms (right) in miR-126&126* versus empty vector-transduced Me665/1 and A375M cell lines. **C)** Representative Real-time PCR analysis of ADAM9 (left), MMP7 (middle) and OPN (right) mRNAs in the A375M cell line. The unresponsive short isoform of ADAM9 mRNA does not carry miR-126&126* binding sites in its 3′UTR. **D)** Relative expression values obtained by western blot analysis of ADAM9 (left), MMP7 (middle) and OPN (right) in A375M cells transfected with oligomers mimicking mature miR-126 or miR-126* *vs* non targeting (no Targ); GAPDH and actin were internal loading controls in RT-PCR and WB, respectively. Columns, of a minimum of two independent experiments; **P<0.001; *P<0.05.

Searching for proteins predicted as putative targets of miR-126&126*, we utilized a bioinformatics-based approach (TargetScan, PicTar and RNAhybrid). Among a number of options, we focused on ADAM9, MMP7 and osteopontin (OPN) for their important role in melanoma progression [16], [18], [19]. Growth and dissemination of human melanoma require a cascade of cellular processes including cellular interactions and proteolytic cleavage of growth factor ligands and extracellular matrix proteins. ADAM9, MMP7 and OPN playing proteolytic, migratory and adhesive functions have a pivotal role in this process [15], [20], [21]. We initially analyzed the expression level of these proteins in cell lysates obtained from differently staged melanoma cell lines. Western blot analysis showed a barely or not detectable signal in normal melanocytes and a direct correlation of their expressions with tumor progression (Figure 5A). To determine whether these genes are biological targets of miR-126&126*, the expression levels of ADAM9, MMP7 and OPN were evaluated as a consequence of either miR-126&126* enforced expression or abrogation. As possibly expected, increased miR-126&126* in Me665/1 and A375M cell lines reduced the expressions of all these three proteins (Figure 5B, left and middle). In addition, all these three proteins exist as secreted fractions promoting motility and invasion of tumor cells [22]–[24]. Soluble ADAM9, MMP7 and OPN, analyzed in the conditioned media from control and miR-transduced metastatic melanoma cells, confirmed their miR-126&126*-dependent reduction (Figure 5B, right). As an interesting validation, no miR-126&126* dependent effects were observed on the 16 kDa isoform of ADAM9 whose 3′ untranslated region (3′ UTR) does not carry miR-126&126* binding sites (Figure 5B, left). qRT-PCR analysis performed in the A375M cell line showed that miR-126&126* play their repressive effects on ADAM9, MMP7 and OPN also at mRNA level (Figure 5C). Moreover, treatment of primary or metastatic melanoma cells with anti-miR-126 and/or 126* LNA oligonucleotides for 48 hours showed a significant increase in the levels of ADAM9 and MMP7 compared with negative control-treated cells as judged by western blot analysis. No significant upmodulation was obtained for OPN (Figure 3 and data not shown).

Searching for independent functions possibly played by miR-126 and miR-126*, we also investigated the expression of ADAM9, MMP7 and OPN proteins in A375M transfected with dsRNAs mimicking either mature miR-126 or miR-126*. Western blot analysis showed that both microRNAs were able to inhibit these putative target genes compared to negative controls (Figure 5D). In particular, miR126* seems to be more effective than miR-126 on ADAM9 regulation (Figure 5D left).

### miR-126 and 126* Directly Regulate ADAM9 and MMP7 Target Genes

**Figure 6:**
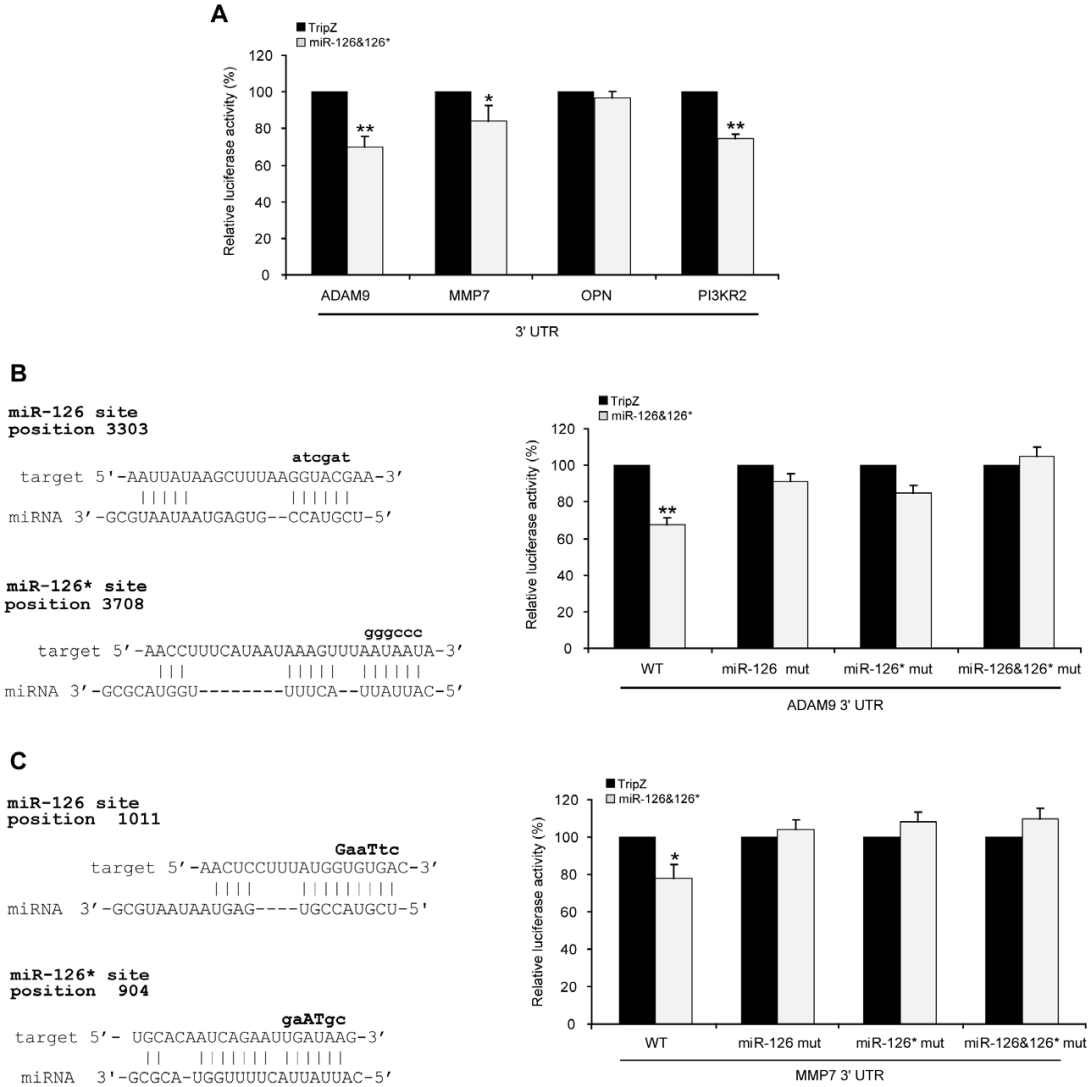
Luciferase Assays of miR-126&126* target genes. **A)** Luciferase reporter assays performed by transfecting a Luc reporter gene (psiCHECK2) linked to 3′-UTR of ADAM9 or MMP7 or OPN or PI3KR2 in miR- versus empty vector-transduced A375M cell lines. **B)** Schematic presentation of predicted miR-126 and miR-126* target sites identified in the ADAM9 3′UTR (left) and relative miR-126&126*-dependent luciferase activities (right) in presence of wild-type (WT) or mutant (mut) binding sites in the-ADAM9 3′UTR. **C)** The same schematic representation (left) and luciferase experiments (right) carried out on MMP7 3′UTR. Columns, of minimum of 5 experiments per group; **P<0.001; *P<0.05.

To verify whether the above reported genes were direct targets of miR-126&126*, we performed a series of reporter luciferase assays by cloning their 3′UTRs downstream of the Renilla open reading frame in the pSiCheck vector. The reporter constructs were transfected into the A375M melanoma cell line stably transduced with miR-126&126* or with the empty vector control. As shown (Figure 6A), co-transfection experiments with the wild-type 3′UTR regions of ADAM9 and MMP7 showed a significant inhibition of the luciferase activity, thus confirming the direct targeting of miR-126&126*. On the contrary, the lack of effects on the OPN 3′UTR supported the option of a miR-126&126* indirect regulation. The secondary regulation of OPN respect to the direct repression of ADAM9 and MMP7 was corroborated by the lack of OPN induction in anti-miR126&126* LNA treated melanomas (Figure 3 and data not shown). In these assays was included the analysis of the 3′ UTR of PI3KR2, recently demonstrated as a direct target of miR-126 [25] (Figure 6A).

**Figure 7:**
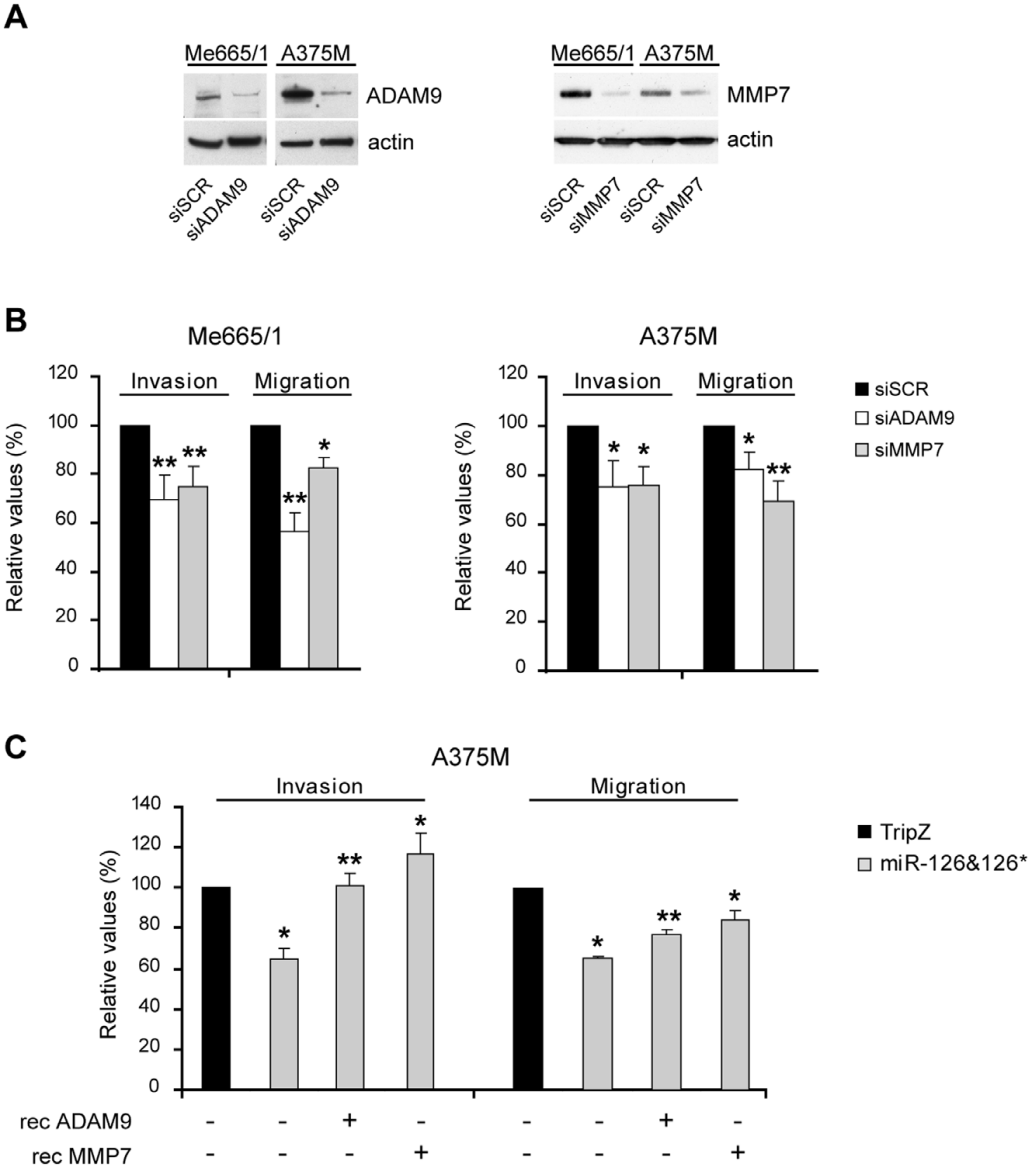
ADAM9 and MMP7 functional roles. A) Western blot analyses showing the effectiveness of stable si-ADAM9, si-MMP7 and scrambled control (SCR) transduction. **B)** Invasion and migration assays in si-ADAM9- or si-MMP7-infected melanoma cell lines compared with scrambled control. **C)** Invasion (left) and migration (right) in presence of either ADAM9 or MMP7 recombinant proteins in miR-126&126*-transduced A375M cell lines compared with control cells. Columns, mean±SD of a minimum of two independent experiments; **P<0.001; *P<0.05.

As a control of specificity, mutations inserted in the binding sites for miR-126&126* in both 3′UTRs restored the luciferase levels (Figure 6B, C). To investigate the actual contribution of ADAM9 and MMP7 modulation to miR-126&126*-dependent outcomes, we tested the effects deriving from separately knocking-down these two genes in A375M and Me665/1 melanoma cells. To this end, cells were stably infected by using a retroviral vector expressing si-ADAM9 or si-MMP7 as well as negative controls (Figure 7A). In both cases we observed a significant reduction of the invasive and chemotactic capabilities of siRNA expressing cells, but not in si-scrambled transduced controls (Figure 7B). The causal relationship between ADAM9 and MMP7 silencing and these functional effects was corroborated by the partial or complete rescues obtained when in the A375M the experiments were performed in presence of ADAM9 or MMP7 recombinant proteins (Figure 7C).

### miR-126&126* Restored Expression Impairs the Proteolytic Activation of Heparin-binding EGF-like Growth Factor (HB-EGF)

**Figure 8:**
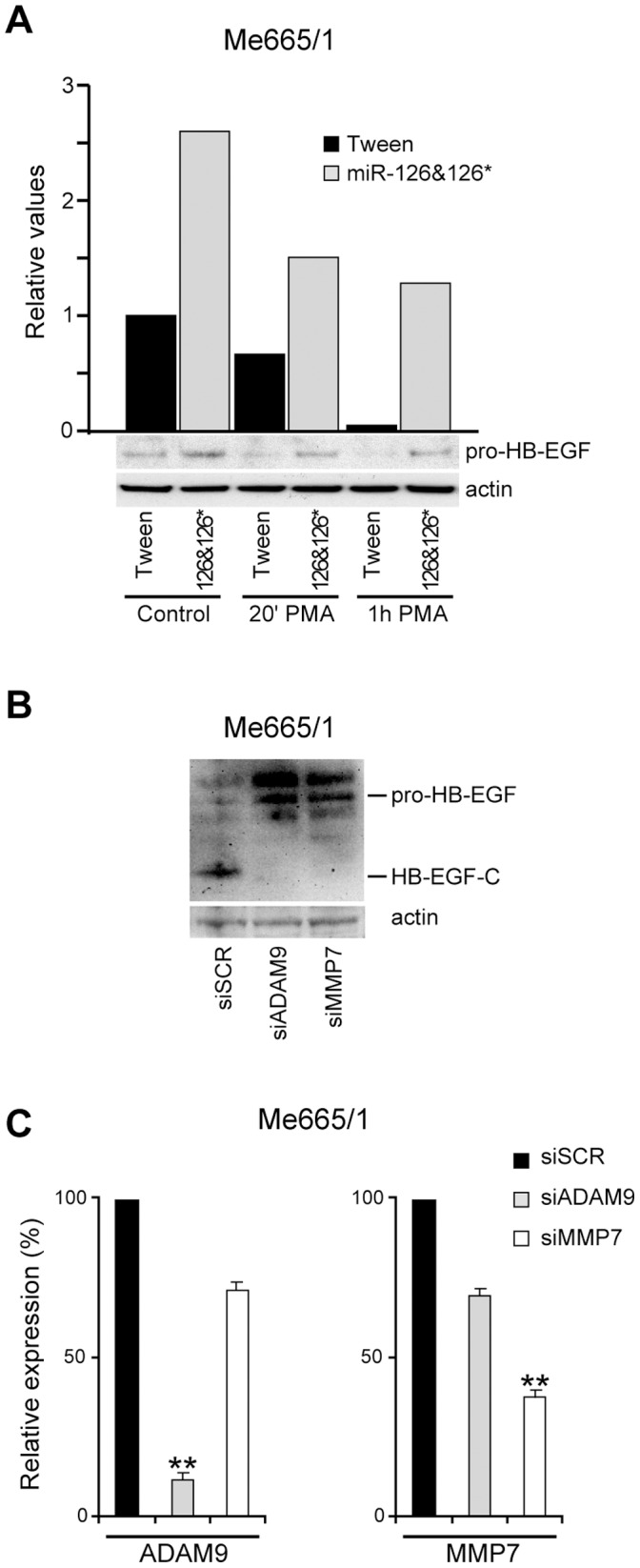
HB-EGF regulation in miR-126&126*-transduced cells. Representative WB of **A)** pro-HB-EGF (bottom) and relative densitometric analysis (top) in miR-126&126*- versus empty vector-transduced Me665/1 melanoma cell line treated or not with PMA. **B**) pro-HB-EGF and HB-EGF-C levels in si-ADAM9- or si-MMP7-infected melanoma compared with si-scrambled control. **C)** Real time PCR analysis of ADAM9 and MMP7 in the same silenced cells.

ADAM9 as well as MMP7 are known to activate HB-EGF through the proteolytic cleavage of the transmembrane pro-HB-EGF precursor, yielding HB-EGF, the soluble ligand of the epidermal growth factor receptor (EGFR), and HB-EGF-C, an intracellular carboxy-terminal fragment [12], [13]. While the soluble fraction is known to bind and activate the intracellular cascade of signaling downstream to EGFR promoting the growth of normal and neoplastic cells [12], [13], HB-EGF-C has been shown to translocate into the nucleus after exposure to shedding stimuli. In melanoma cells the low or absent levels of miR-126&126*, unblocking ADAM9 and MMP7, should result in an increased activation of pro-HB-EGF, consequently producing a higher quantity of HB-EGF-C translocation into the nucleus [26]. Accordingly, we observed a significant accumulation of HB-EGF precursors in miR-126&126*-transduced Me665/1 cells (Figure 8A) in association with the downregulation of the soluble active forms of ADAM9 and MMP7 (Figure 5B right). In addition, different kinetics of HB-EGF proteolytic activation were observed in melanoma cells, transduced or not with miR126/126*, after treatment with the phorbol ester PMA. As shown (Figure 8A), no more HB-EGF precursors were detectable after 1 hr of PMA treatment in empty-vector control cells, thus indicating the complete cleavage of pro-HB-EGF. On the contrary, the enforced expression of miR-126&126*, and the consequential inhibition of ADAM9 and MMP7, only allow an early boost of PMA-dependent activation, thereafter maintaining a steady state level of pro-HB-EGF.

We then evaluated the activation of HB-EGF in PMA-treated Me665/1 metastatic cell line after ADAM9 and MMP7 separate silencing. Interestingly, we observed a strong reduction of HB-EGF activation paralleled by precursor accumulation in either si-ADAM9 or si-MMP7 transduced cells compared with a si-scrambled control, thus confirming a major proteolytic function of these two metalloproteases among the great number of miR-126&126* regulated targets (Figure 8B). As the sole abrogation of ADAM9 or MMP7 appeared sufficient to strongly inhibit their proteolytic action, we might consider the hypothesis of a cross regulation between MMP7 and ADAM9. qRT-PCR indicated this relationship in si-ADAM9 and si-MMP7 transduced cells (Figure 8C).

### Global mRNA Analysis of A375M and Me665/1 Overexpressing miR-126&126*

Based on our results showing the involvement of miR-126&126* in several oncogenic pathways, we decided to look at miR-126&126* global molecular effects in metastatic melanoma cells. We then realized mRNA microarray analysis of A375M and Me665/1 by comparing miR-126&126*- with empty vector-transduced control cells (see Materials and Methods for the accession number and details). The comparative analyses of miR-126&126*- associated signatures showed a total of 4300 and 4491 modulated genes in Me665/1 and A375M cell lines respectively, including 1229 overlapping genes. The intersection between Target Scan list of miR-126&126* putative targets and genes differentially expressed in each melanoma cell line, evidenced 14 and 12 common genes for Me665/1 and A375M, respectively (Table S1, [50]–[53]). Among them, only four miR-126&126* putative targets were shared by both melanomas (see Table S1) being two of them, the solute carrier family 7, member 5 (SLC7A5) [6] and ADAM9 ([27] and this paper) already confirmed as direct targets of miR-126. These results support the representativeness of our arrays, strongly suggesting the two additional targets, the guanine nucleotide binding protein, alpha 13, (GNA13), required for invasion and dissemination of metastatic melanoma [28], and the ribosomal RPL27A, involved in p53 regulation [29], as good candidates for future studies.

**Figure 9:**
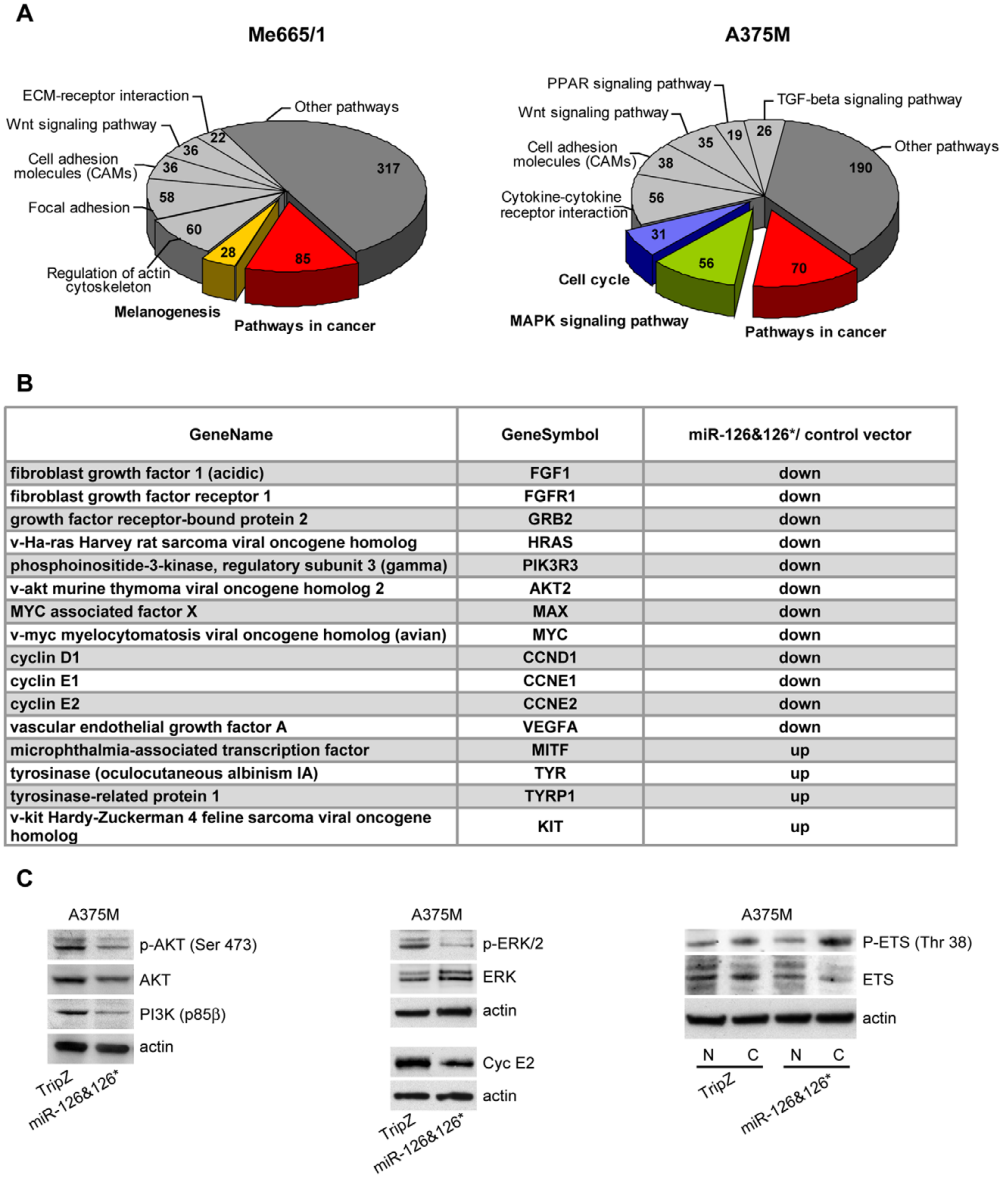
Gene expression profiles. A) KEGG pathway analysis of miR-126&126* associated molecular signatures deriving from Me665/1 and A375M cell lines transduced with miR-126&126* and compared with controls. Up- and down- differentially expressed genes correspond to >2 and <2, respectively. **B)** A short list of functionally relevant genes modulated by miR-126&126* in Me665/1 and/or A375M. **C**) WB validation of some selected genes.

We then identified the pathways differentially expressed by a functional class scoring analysis, using the David software applied to the gene lists of Me665/1 and A375M cell lines. This analysis, based on the Kyoto Encyclopedia of Genes and Genomes (KEGG) annotations, identified a number of pathways significantly modulated by miR-126 and 126* (Figure 9A).

The most modulated ones, including the highest number of differentially expressed genes, were markedly involved in oncogenic transformation, cell cycle and signaling. We mainly focused on “Pathways in cancer”, “MAPK signaling”, “Cell cycle” and “Melanogenesis”, including several targets directly altered by miR-126&126*, as PI3K, and a high number of indirectly regulated genes, functionally associated with melanoma induction and/or progression (Figure 9B). Genes pertaining to “Pathways in cancer” and specifically modulated in A375M and/or Me665/1 melanoma cell lines are listed (Table S2) and highlighted in their functional positions (Figures S2 and S3).

In addition the array results of Me665/1 cells showed the involvement of the “Melanogenesis” pathway (Figure S4 and Table S3) confirming the upregulation of MITF, TYR and TRP1, involved in the production of melanin [30]. Some representative results were confirmed by Western blot analysis (Figure 9C). As shown, we observed a miR-126&126*-dependent reduction of ERK1/2 activating phosphorylation, paralleled by downregulation of nuclear ETS1, either total or phosphorylated (P-Thr38), and delocalization of its activated fraction into the cytoplasm. Moreover, we confirmed the decrease of total and P-(Ser473) Akt and that of cyclin E2.

## Discussion

Malignant melanoma represents a clinical challenge as current therapeutic treatments are greatly inadequate. At present the only effective therapy is the surgical resection of early detected lesions.

A promising reservoir of biomarkers as well as of putative therapeutic targets is now represented by the microRNA family, a class of small non coding regulatory RNAs mostly displaying their functions at post-transcriptional levels [31]. In the last few years microRNAs have been demonstrated as important players in all the main biological processes, including tumorigenesis, where they can act either as oncogenes or tumor suppressors [32].

We focused our studies on miR-126&126* as they were the most downmodulated microRNAs in a panel of differently staged melanoma cell lines compared with normal human melanocytes analyzed by microRNA profiling (Figure 1A). We confirmed the down-regulation of endogenous miR-126&miR-126* associated with melanoma progression (Figure 1B), with a significant reduction between primary and advanced melanomas, demonstrating the antineoplastic role deriving from their restored expression *in vitro* as well as *in vivo* (Figures 2, 3 and 4). Trying to generalize miR-126&126* function, as recipients of miR transduction we selected two metastatic melanoma cell lines characterized by a different mutational status of BRAF and NRAS (i.e. BRAF V600E and NRAS wt for A375M and BRAF wt and NRAS Q61R for Me665/1) [33], [34].

Looking for miR-126&126* predicted target genes, we selected ADAM9, MMP7 and OPN proteins based on bioinformatics analysis and on their functional role in melanocyte transformation. These three proteins are involved in biological processes like cell-cell and cell-matrix interaction, intracellular signaling and proteolysis. Although playing their function in normal tissue remodeling, their overexpression has been associated with a wide variety of cancers, including melanoma [16], [18], [19]. In this report, we demonstrated that ADAM9 and MMP7 were direct targets of miR-126&miR-126*, whereas they only indirectly regulated OPN (Figure 6), possibly through an auto-sustaining loop linking OPN and MMP7 [23].

Beside a number of functions, ADAM9 and MMP7 share the proteolytic cleavage of HB-EGF [12], [13], whose expression results significantly increased in many human cancers. In melanoma and other cancer cells, HB-EGF was found to be the predominant EGFR ligand, thus suggesting its potential therapeutic value [35]. While the N-terminal region (secreted HB-EGF) has been extensively studied as a ligand of the EGF receptor and activator of MAPK and PI3K/Akt pathways [36], the C-terminal region (HB-EGF-C) has been reported to enter the nucleus where it binds and delocalizes a number of genes critical for cell proliferation [37]. Here we demonstrate that the restored expression of miR-126&126* interferes with the activating cleavage of the HB-EGF precursor (Figure 8A) and that a specific silencing of either ADAM9 or MMP7 is sufficient to reduce the activation of HB-EGF (Figure 8B). According to the functional crosstalk reported between other members of the MMP and ADAM families, showing a MMP7-dependent transcriptional and post-transcriptional activation of ADAM12 and ADAM28 [38], [39] as well as ADAM17 targeting of MMP-2 and MMP-9 [40], it is reasonable to speculate a regulatory connection also between MMP7 and ADAM9 in melanoma. Accordingly, silencing results showed a partial ADAM9 reduction in siMMP7 treated cells and viceversa (Figure 8C). Altogether, we may assess that in normal melanocytes the presence of miR-126&126* tightly regulates the levels of ADAM9 and MMP7, thus limiting the amount of pro-HB-EGF shedding and in turn the activation of proliferative and anti-apoptotic pathways. Conversely, the low levels of miR-126&126* expressed in melanoma are incompetent to prevent the ectodomain shedding and activation of HB-EGF, thus inducing several pro-neoplastic signals.

In addition, gene profile results gave us a broader view supporting the functional involvement of miR-126&126* in some key pathways of the oncogenic transformation, above all PI3K/AKT and RAS/RAF/MEK/ERK. The contemporary activation of these two signaling pathways, frequently activated in cancer, including melanoma through genetic alterations [41], supports the implication of combined therapeutics against both pathways [42], a role theoretically played by miR-126&126* (Figure 9, Figures S2 and S3). The possible anti-metastatic role of miR-126&126* was shown by the repression of crucial molecules involved in the MAPK (i.e. FGF/FGFR, EGF and Ras), in the PI3K pathway (i.e. PI3K and Akt) and in the cell cycle regulation (i.e. cyclin D1, E1, E2) (Figure 9C). Interestingly, the rise of c-KIT, MITF and TRP1 indicated the re-induction of differentiation and melanogenesis (Figure S4 and Table S3), which is more evident in Me665/1 than in A375M, possibly because of their different advancement grade.

It is finally important to mention the role played by miR-126 in endothelial cells, where it was described to suppress cancer-mediated endothelial recruitment in metastatic breast cancer. Png and coauthors suggested that most of the suppressive effect displayed by miR-126 might be secondary to endothelial cell function in metastatic initiation through miR-126 targeting of the pro-angiogenic genes IGFBP2, PITPNC1 and MERTK [9].

In conclusion, miR-126&126* downregulation appears as a key event in melanoma progression. The lack of miR-126&126*, to some extent relaying on ADAM9 and MMP7 through HB-EGF activation, appears to converge into several other oncogenic pathways according to a more wide-ranging regulatory role, as demonstrated by gene profiling studies (Figures S2, S3 and S4). In view of this, and considering the low toxicity expected from the re-expression of physiological microRNAs, the replacement of miR-126 and miR-126* might be suggested as a therapeutic option for the treatment of advanced melanoma, possibly in combination with more traditional drugs.

## Materials and Methods

### Cell Lines and Transduction

Human melanoma cell lines were obtained and characterized as previously described [17]. Normal human epidermal melanocytes from foreskin were obtained from Promocell (Heidelberg, Germany). The early passage melanoma cells were kindly provided by Dr S. D’Atri (IDI IRCCS, Rome, Italy). Two cell lines were obtained from primary tumors (GR-mel, ST-mel) and three from lymph node metastases (CR-mel, CN-mel, MR/C-mel) [43].

A 604-base-pair fragment spanning the miR-126&126*locus was PCR-amplified from human genomic DNA (NT_024000.16 from nt 347834 to nt 348437) by using AccuPrime Taq DNA polymerase high fidelity (Invitrogen, Carlsbad, CA, USA). This miR-126&126* containing fragment, after sequencing, was cloned in the pCR 2.1 vector (Invitrogen) and thereafter inserted under the CMV promoter into EcoR1 site of a variant third-generation lentiviral vector, pRRL-CMV-PGK- GFP-WPRE, called Tween [44], or into the EcoR1 site of the doxycycline inducible lentiviral vector, TripZ (Open Biosystem, Huntsille, AL, USA). In the latter the induction efficiency, obtained with 2 µg/ml of doxycycline, was evaluated 48 h and 72 h after treatment. Vector- and miR-126&126*-transduced A375M and Me665/1 bulk cultures as well as cellular clones, obtained by limiting dilutions, were utilized.

### All the Utilized Primers are Listed in Table S4

#### Real time qRT-PCR

Real time quantification was performed according to the TaqMan technology (Applied Biosystems, Foster City, CA, USA): miR-126 #002228; miR-126* #000451; RNU6B (#001093); ADAM9 (#Hs00177638_m1), MMP7 (#Hs01042796_m1) and GAPDH#4326317E).

#### Gene silencing

ADAM9 and MMP7 silencing was performed by using 4 unique 29-mer shRNA constructs in retroviral GFP vector, #TG314947 for ADAM9, #TG311438 for MMP7 and #TR30013 for scrambled negative control (OriGene Technologies, Rockville, MD, USA).

miRCURY locked-nucleic-acid (LNA) Power Inhibitor knockdown probes for miR-126 and -126* were obtained from Exiqon (Copenhagen, Denmark), (Product numbers #426717-00 and #426718-00). Anti-miRNAs (anti-miRs) were used at 200 nM. When anti-miR-126 and anti-miR-126* were used together, they were pooled at equal molarities (final concentration of each, 100 nM; total concentration, 200 nM). In all the experiments a LNA microRNA Inhibitor Negative Control was utilized as a comparison (#199004-00). Transfections were performed using Fugene HD according to the manufacturer’s instructions (Promega Corporation, Madison, WI, USA).

#### Western blot

Was performed according to standard procedures. Antibodies against ADAM9, HB-EGF (Santa Cruz Biotechnology, Santa Cruz, CA,USA); MMP7 (R&D Systems, Minneapolis, MN, USA), OPN, PI3KR2, CycE2 (Abcam, Cambridge, MA, USA), AKT, P-AKT (Ser453), ERK1/2 (Cell Signaling, Danvers, MA, USA), P-ERK1/2 (Immunological Science, Rome, Italy), ETS-1 (Novocastra, Newcastle Upon Tyne, UK), ETS-1 (PThr38) (Biosource, Camarillo, CA, USA) were used in accordance to the manufacturer’s instructions. ACTIN (Oncogene Research, La Jolla, CA, USA) was used as a loading control and for subsequent quantification. The expression levels were evaluated by the AlphaView or Image Quant Software.

#### Luciferase assay

Luciferase reporter assays were performed as reported [45]. Briefly, the 3′UTRs of target genes (ADAM9: NM_003816 from nt 3164 to nt 3860, MMP7: NM_002423 from nt 820 to nt 1070, OPN: NM_000582 from nt 1046 to nt 1535, PI3KR2: NM_005027 from nt 3759 to nt 3958) predicted to interact with miR-126&126*, were PCR amplified (see Table 4 for primer sequences) and cloned in psiCHECKTM-2 vector (Promega). For the luciferase reporter assay, each psiCHECKTM-3′UTR plasmid was transfected in empty- or miR-126&126*-vector transduced A375M cell line. A375M empty vector-transduced values were considered as 100%. As controls of specificity, point mutations were inserted in the wild type seed binding sequence. Mutated nucleotides are indicated in Figure 6.

### In vitro Functional Assays

Functional studies were performed as previously described [17]. The proliferative rate of melanoma cells was evaluated by a colorimetric assay XTT-based (Roche Molecular Biochemicals, Mannheim, Germany) and quantified using an ELISA plate reader (VICTOR2, Perkin Elmer). Migration was assayed, as previously described using uncoated cell culture inserts (Corning Costar Corporation, Cambridge, MA, USA) with 8 µm pores [46]. A total of 5×10^4^ cells were placed in the upper compartment in 100 µl of Dulbecco’s Modified supplemented with 10% fetal bovine serum (FBS), whereas 600 µl of DMEM supplemented with 10% fetal bovine serum (FBS) were placed into the lower compartment of the chamber as a chemoattractant. For invasion studies, membranes were coated with 100 µg/cm^2^ of Matrigel growth factor reduced (Becton Dickinson, Bedford, MA, USA) as a barrier. All these assays were incubated at 37°C in 5% CO2. After 24–48 hr, the cells attached to the upper side of the membrane were removed with a cotton swab, each membrane was fixed and stained with crystal violet solution [47]. Chemotaxis and invasiveness were evaluated by a colorimetric assay at 595 nm in a microplate reader (Wallac VICTOR2, Turku, Finland) by counting the relative number of cells on the undersurface of the membrane. The data were expressed as the mean absorbance ± SD for triplicate wells. The statistical analysis was performed by Student’s t test in all the reported experiments.

The same procedures were used for miR-126&126* overexpressing or stably transduced scrambled control or si-ADAM9 or si-MMP7 cell lines. For the assays conducted in presence of human recombinant proteins, ADAM9 (R&D) or MMP7 (Prospec, Ness-Ziona, Israel) were applied to the transwell chambers (2 µg/ml).

Apoptosis was measured by flow cytometry and cells with a sub-G0 content identified as apoptotic. Caspase assays were performed with Apo-ONE® Homogeneous Caspase-3/7 Assay (Promega).

### Growth in Semisolid Medium

Base layers of complete DME medium containing 0.5% agar were set in 60-mm plastic dishes. The bottom agar was overlaid with 1.5 ml of 0.3% agar containing 10^4^ cells. Cultures were incubated for 4–6 weeks at 37°C and the colonies counted using an inverted microscope. Three experiments were performed for each cell line and results calculated as the average±SD of 3 dishes for each condition.

#### *In vivo* assays

Empty vector or miR-126&126*-transduced Me665/1 or A375M cells were injected subcutaneously or intra-venom at the dose of 10^6^ cells in adult athymic nude mice (minimum n = 5 mice per group-Charles River, Calco, Italy). Tumor growths were monitored by caliper measuring. For dissemination studies mice were subjected to doxycycline treatment (2 mg/ml in water drink) until the end of experiment (usually 6 wks from inoculum). Doxycycline induction was monitored by detecting the red fluorescent protein (RFP) reporter after tumor mass dissociation. Lung and liver metastases were counted under a stereomicroscope. For lung metastasis count, India ink perfusion via windpipe was used. Mice were treated and maintained at the Istituto Superiore di Sanità (Rome, Italy) according to the Italian institutional guidelines.

#### Whole human genome expression detection by oligo microarray

To analyze the global mRNA expression in Me665/1/Tween *vs* Me665/1/miR-126&126* and A375M/TripZ *vs* A375M/miR-126/126* cell lines, RNAs were hybridized on Agilent whole human genome oligo microarray (#G4112F, Agilent Technologies, Palo Alto, CA, USA). Microarray data conforming to the MIAME guidelines have been deposited in ArrayExpress http://www.ebi.ac.uk/arrayexpress under the accession number E-MEXP-3629.

#### Microarray data analysis

Microarray results were analyzed by using the GeneSpring GX 11 software (Agilent Technologies) as previously reported [48]. Differentially expressed genes, selected as having a 2-fold expression difference between their geometrical mean in two groups of interest, were employed for Cluster Analysis of samples, using the Manhattan correlation as a measure of similarity.

The **D**atabase for **A**nnotation, **V**isualization and **I**ntegrated **D**iscovery (**DAVID** ) v6.7 has been utilized to identify enriched functional-related gene groups and visualize genes on KEGG pathway maps [49].

#### Target analysis

Bioinformatic analysis was performed by using these specific programs: TargetScan 6.2 (http://www.targetscan.org/), PicTar (http://pictar.bio.nyu.edu/) and RNAhybrid (http://bibiservice.techfak.uni-bielefeld.de/).

#### Statistical analysis

Statistical and frequency distribution analysis was performed by Excel. Differences between two or three groups were compared with Student’s t-test. A value of p<0.05 or less was considered to be statistically significant.

## Supporting Information

Figure S1 MiR-126 and miR-126* **overexpression.** Ectopic miR-126 and miR-126* levels evaluated by qReal-time PCR in either constitutively (Me665/1, left) or doxicyclin inducible (A375M, right) transduced melanoma cell lines. In the inducible system the analysis was performed at 48 and 72 hours after treatment.
(PDF)

Figure S2 **Schematic model from KEGG “Pathways in cancer”.** Genes differentially modulated, either up (log_2_ ratio >2) or down (log_2_ ratio <2), by miR-126&126* in Me665/1 melanoma cell line are highlighted in red.
(DOCX)

Figure S3 **Schematic model from KEGG “Pathways in cancer”.** Genes differentially modulated, either up (log_2_ ratio >2) or down (log_2_ ratio <2), by miR-126&126* in A375M melanoma cell line are highlighted in red.
(DOCX)

Figure S4 **Schematic model from KEGG “Melanogenesis”.** Genes differentially modulated, either up (log_2_ ratio >2) or down (log_2_ ratio <2), by miR-126&126* in Me665/1 melanoma cell line are highlighted in red.
(DOCX)

Table S1 Identification of miR-126&126* direct target genes validated by microarray among those reported by Target Scan. These lists represent the intersections of miR-126&126* direct target genes validated by microarray among those reported by Target Scan as putative miR-126-3p targets. Genes highlighted in red are shared by Me665/1 and A375M melanoma cell lines. Genes in bold derive from the list reported by Target Scan of targets conserved across most mammals.
(DOCX)

Table S2 **Pathways in cancer.** Results of Me665/1 and A375M melanoma cell lines transduced with miR-126&126* compared with controls. This list of differentially expressed genes derives from KEGG “Pathways in cancer”. The differential gene expression is obtained as log2 of the ratio between miR-126&126* and control cells. Up- and down-regulated genes correspond to >2 and < 2, respectively.
(DOCX)

Table S3 **Melanogenesis.** Results of Me665/1 melanoma cell line transduced with miR-126&126* compared with controls. This list of differentially expressed genes derives from KEGG “Melanogenesis”. The differential gene expression is obtained as log2 of the ratio between miR-126&126* and control cells. Up- and down-regulated genes correspond to >2 and < 2, respectively.
(DOCX)

Table S4 **Primer list.** This list includes all the utilized primers for miR-126&126* and 3′UTRs cloning and for semiquantitative RT-PCR.
(DOCX)
