# Supplementary figures and images for: miR-126&126* Restored Expressions Play a Tumor Suppressor Role by Directly Regulating ADAM9 and MMP7 in Melanoma
Source: PLoS One. 2013 Feb 21;8(2):e56824. doi: 10.1371/journal.pone.0056824 (PMC3578857; doi:10.1371/journal.pone.0056824)

**Me665/1**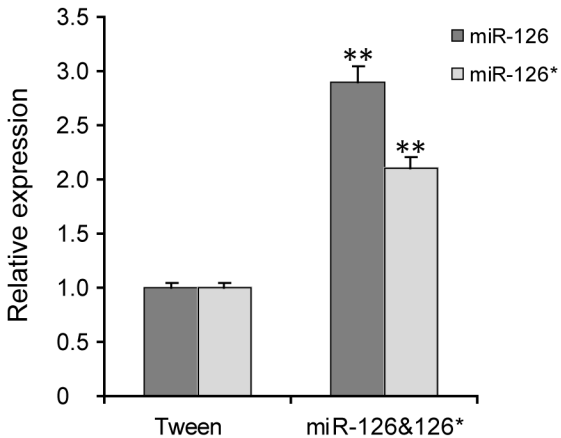**A375M**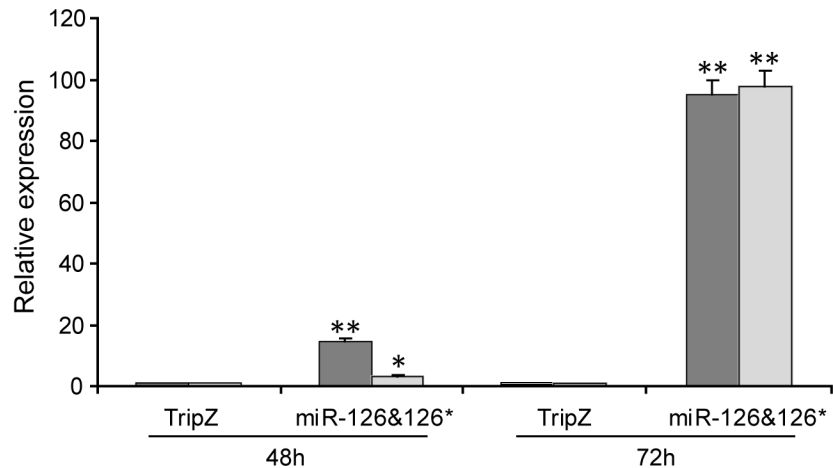**Figure S1**

Supplement: Figure S1 — MiR-126 and miR-126* overexpression. Ectopic miR-126 and miR-126* levels evaluated by qReal-time PCR in either constitutively (Me665/1, left) or doxicyclin inducible (A375M, right) transduced melanoma cell lines. In the inducible system the analysis was performed at 48 and 72 hours after treatment. (PDF) [file pone.0056824.s001.pdf]

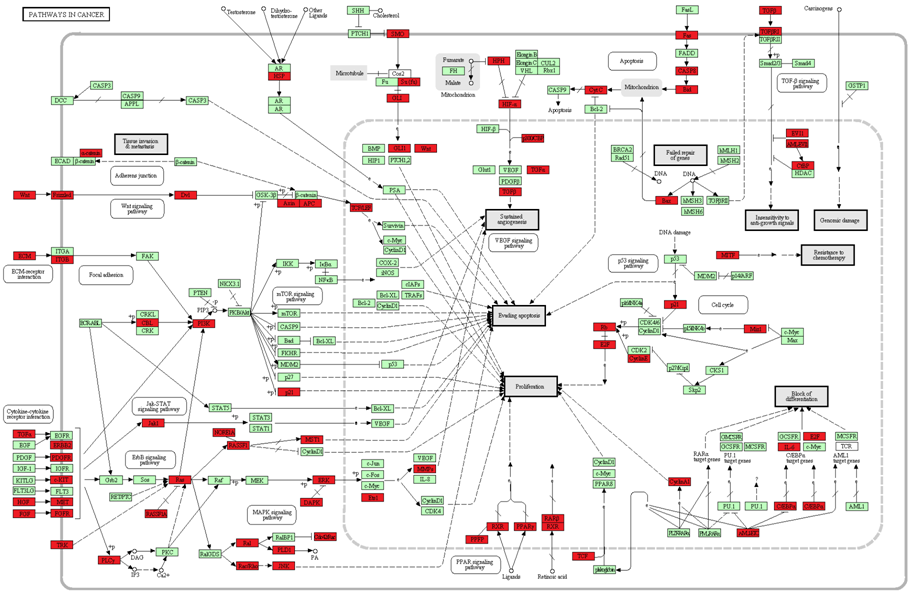


**Figure S2**

Supplement: Figure S2 — Schematic model from KEGG “Pathways in cancer”. Genes differentially modulated, either up (log2 ratio >2) or down (log2 ratio <2), by miR-126&126* in Me665/1 melanoma cell line are highlighted in red. (DOCX) [file pone.0056824.s002.docx]

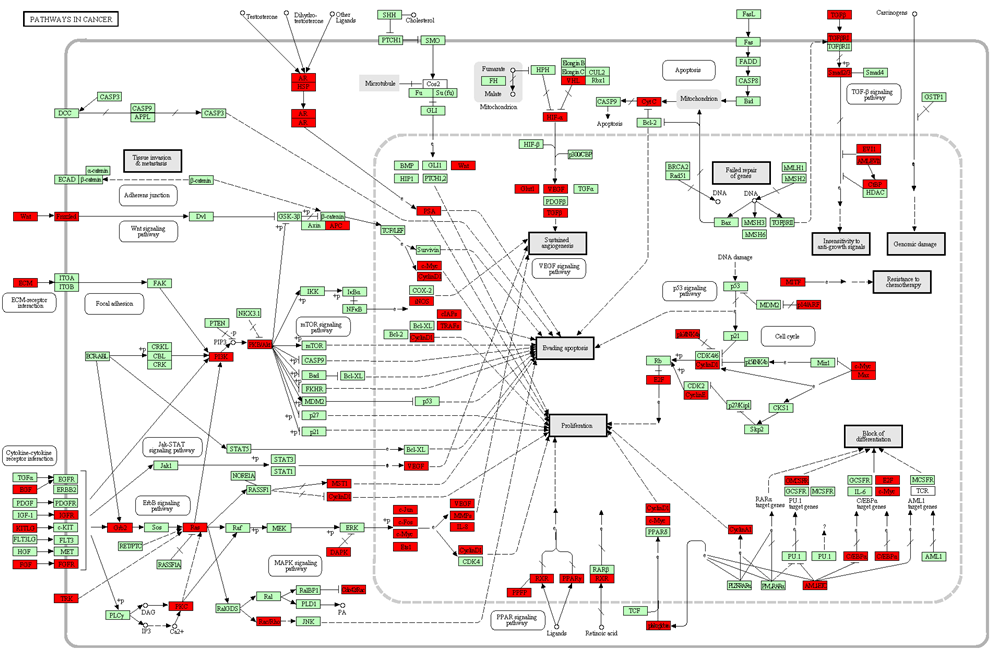


**Figure S3**

Supplement: Figure S3 — Schematic model from KEGG “Pathways in cancer”. Genes differentially modulated, either up (log2 ratio >2) or down (log2 ratio <2), by miR-126&126* in A375M melanoma cell line are highlighted in red. (DOCX) [file pone.0056824.s003.docx]

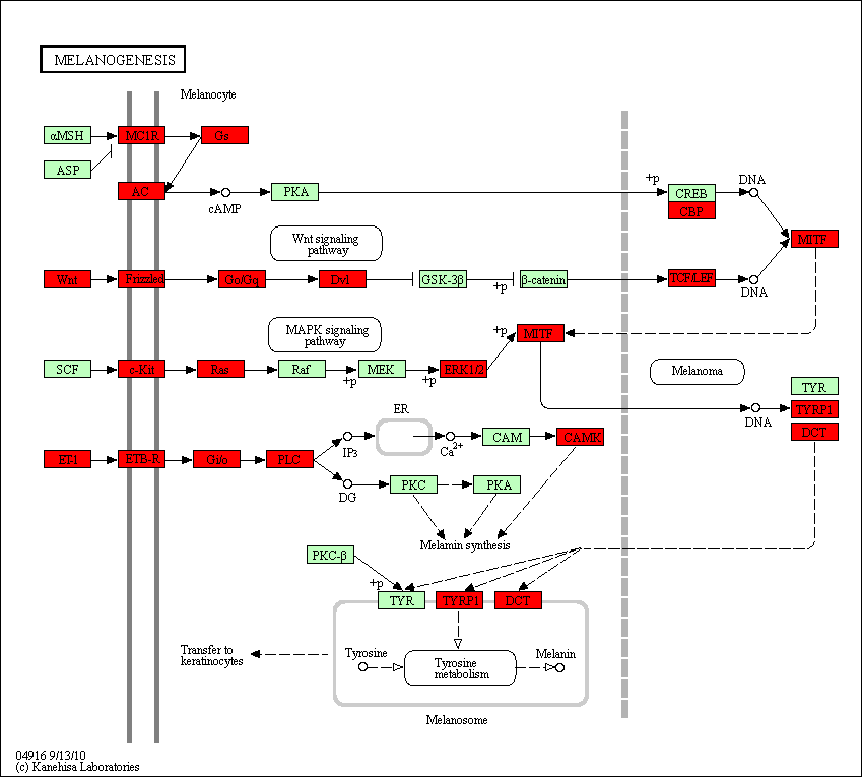


**Figure S4**

Supplement: Figure S4 — Schematic model from KEGG “Melanogenesis”. Genes differentially modulated, either up (log2 ratio >2) or down (log2 ratio <2), by miR-126&126* in Me665/1 melanoma cell line are highlighted in red. (DOCX) [file pone.0056824.s004.docx]
