# Supplementary material for: miR-126&126* Restored Expressions Play a Tumor Suppressor Role by Directly Regulating ADAM9 and MMP7 in Melanoma
Source: PLoS One. 2013 Feb 21;8(2):e56824. doi: 10.1371/journal.pone.0056824 (PMC3578857; doi:10.1371/journal.pone.0056824)
Supplement: Table S1 — Identification of miR-126&126* direct target genes validated by microarray among those reported by Target Scan. These lists represent the intersections of miR-126&126* direct target genes validated by microarray among those reported by Target Scan as putative miR-126-3p targets. Genes highlighted in red are shared by Me665/1 and A375M melanoma cell lines. Genes in bold derive from the list reported by Target Scan of targets conserved across most mammals. (DOCX) [file pone.0056824.s005.docx]

| **Me665/1** |  |  |  |  |
| --- | --- | --- | --- | --- |
| **GeneSymbol** | **miR vs control** | **Genbank Accession** | **GeneName** | **References** |
| [**PEX5**](http://www.ncbi.nlm.nih.gov/sites/entrez?Db=gene&Cmd=ShowDetailView&TermToSearch=5830) | **down** | [**NM_000319**](http://www.ncbi.nlm.nih.gov/entrez/query.fcgi?cmd=Search&db=nuccore&term==NM_000319) | **peroxisomal biogenesis factor 5** |  |
| RPL27A | down | NM_000990 | ribosomal protein L27a |  |
| SDC2 | down | NM_002998 | syndecan 2 |  |
| **SLC7A5** | **down** | **NM_003486** | **solute carrier family 7 (cationic amino acid transporter, y+ system), member 5** | [6] |
| IRS2 | down | NM_003749 | insulin receptor substrate 2 |  |
| **ADAM9** | **down** | **NM_003816** | **ADAM metallopeptidase domain 9 (meltrin gamma)** | [27] |
| PIK3CD | down | NM_005026 | phosphoinositide-3-kinase, catalytic, delta polypeptide |  |
| **GNA13** | **down** | **NM_006572** | **guanine nucleotide binding protein (G protein), alpha 13** |  |
| **PLK2** | **down** | **NM_006622** | **polo-like kinase 2 (Drosophila)** | **[50]** |
| **PLXNB2** | **down** | **NM_012401** | **plexin B2** |  |
| **CAMSAP1** | **down** | **NM_015447** | **calmodulin regulated spectrin-associated protein 1** |  |
| LARP6 | down | NM_018357 | La ribonucleoprotein domain family, member 6 |  |
| CHST6 | down | NM_021615 | carbohydrate (N-acetylglucosamine 6-O) sulfotransferase 6 |  |
| **THAP6** | **down** | **NM_144721** | **THAP domain containing 6** |  |

| **A375M** |  |  |  |  |
| --- | --- | --- | --- | --- |
| **GeneSymbol** | **miR vs control** | **Genbank Accession** | **GeneName** | **References** |
| VPS53 | down | NM_001128159 | vacuolar protein sorting 53 homolog (S. cerevisiae) |  |
| C5orf47 | down | NM_001144954 | chromosome 5 open reading frame 47 |  |
| RPL27A | down | NM_000990 | ribosomal protein L27a |  |
| SOX2 | down | NM_003106 | SRY (sex determining region Y)-box 2 | [51] |
| **SLC7A5** | **down** | **NM_003486** | **solute carrier family 7 (cationic amino acid transporter, y+ system), member 5** | [6] |
| **ADAM9** | **down** | **NM_003816** | **ADAM metallopeptidase domain 9 (meltrin gamma)** | [27] |
| **IRS1** | **down** | **NM_005544** | **insulin receptor substrate 1** | **[52]** |
| **GNA13** | **down** | **NM_006572** | **guanine nucleotide binding protein (G protein), alpha 13** |  |
| **EFHD2** | **down** | **NM_024329** | **EF-hand domain family, member D2** |  |
| KIAA1715 | down | NM_030650 | KIAA1715 |  |
| **SPRED1** | **down** | **NM_152594** | **sprouty-related, EVH1 domain containing 1** | **[53]** |
| **FBXO33** | **down** | **NM_203301** | **F-box protein 33** |  |

**Table S1**
