# Supplementary material for: miR-126&126* Restored Expressions Play a Tumor Suppressor Role by Directly Regulating ADAM9 and MMP7 in Melanoma
Source: PLoS One. 2013 Feb 21;8(2):e56824. doi: 10.1371/journal.pone.0056824 (PMC3578857; doi:10.1371/journal.pone.0056824)
Supplement: Table S2 — Pathways in cancer. Results of Me665/1 and A375M melanoma cell lines transduced with miR-126&126* compared with controls. This list of differentially expressed genes derives from KEGG “Pathways in cancer”. The differential gene expression is obtained as log2 of the ratio between miR-126&126* and control cells. Up- and down-regulated genes correspond to >2 and < 2, respectively. (DOCX) [file pone.0056824.s006.docx]

| **Gene Symbol** | **Genbank Accession** | **Me665/1**  **miR-126&126* vs Tween** |  |  |  | **Gene Symbol** | **Genbank**  **Accession** | **A375M**  **miR-126&126* vs TripZ** |
| --- | --- | --- | --- | --- | --- | --- | --- | --- |
| **BAX** | NM_138763 | down |  |  |  | **AKT2** | NM_001626 | down |
| **BID** | NM_197966 | down |  |  |  | **AR** | NM_000044 | down |
| **CASP8** | NM_033355 | down |  |  |  | **CCND1** | NM_053056 | down |
| **CBLB** | NM_170662 | down |  |  |  | **CCNE1** | NM_001238 | down |
| **CCNE2** | NM_057749 | down |  |  |  | **CCNE2** | NM_057749 | down |
| **CDKN1A** | NM_078467 | down |  |  |  | **CDC42** | NM_044472 | down |
| **COL4A1** | NM_001845 | down |  |  |  | **CSF2RA** | NM_172249 | down |
| **CTBP1** | NM_001012614 | down |  |  |  | **CTBP1** | AL137653 | down |
| **CYCS** | NM_018947 | down |  |  |  | **DAPK1** | NM_004938 | down |
| **DAPK2** | NM_014326 | down |  |  |  | **E2F1** | NM_005225 | down |
| **DVL3** | NM_004423 | down |  |  |  | **E2F2** | NM_004091 | down |
| **E2F3** | NM_001949 | down |  |  |  | **ETS1** | NM_005238 | down |
| **EP300** | NM_001429 | down |  |  |  | **FGF1** | NM_000800 | down |
| **ETS1** | NM_005238 | down |  |  |  | **FGFR1** | NM_023108 | down |
| **FGF1** | NM_000800 | down |  |  |  | **FZD5** | NM_003468 | down |
| **FGF16** | NM_003868 | down |  |  |  | **FZD8** | NM_031866 | down |
| **FGFR1** | NM_023110 | down |  |  |  | **GRB2** | NM_002086 | down |
| **FZD1** | NM_003505 | down |  |  |  | **HRAS** | NM_005343 | down |
| **FZD5** | NM_003468 | down |  |  |  | **HSP90AA1** | NM_005348 | down |
| **GLI1** | NM_005269 | down |  |  |  | **KITLG** | NM_000899 | down |
| **HRAS** | NM_005343 | down |  |  |  | **LAMA5** | NM_005560 | down |
| **HSP90AB1** | NM_007355 | down |  |  |  | **LAMC2** | NM_005562 | down |
| **IL6** | NM_000600 | down |  |  |  | **MAX** | NM_197957 | down |
| **ITGB1** | NM_133376 | down |  |  |  | **MMP1** | NM_002421 | down |
| **LAMA3** | NM_198129 | down |  |  |  | **MYC** | NM_002467 | down |
| **LAMA4** | NM_002290 | down |  |  |  | **NOS2** | NM_000625 | down |
| **LAMB3** | NM_001017402 | down |  |  |  | **PIK3R3** | NM_003629 | down |
| **MAPK3** | NM_002746 | down |  |  |  | **PPARG** | NM_138711 | down |
| **MAPK9** | NM_002752 | down |  |  |  | **PPARGC1B** | NM_133263 | down |
| **MET** | NM_000245 | down |  |  |  | **RUNX1T1** | NM_004349 | down |
| **MMP2** | NM_004530 | down |  |  |  | **RXRA** | AK090416 | down |
| **PDGFRB** | NM_002609 | down |  |  |  | **SLC2A1** | NM_006516 | down |
| **PIAS2** | NM_173206 | down |  |  |  | **STK4** | NM_006282 | down |
| **PIK3CD** | NM_005026 | down |  |  |  | **TGFB3** | NM_003239 | down |
| **PLD1** | NM_002662 | down |  |  |  | **TPM3** | NM_001043352 | down |
| **PPARG** | NM_138711 | down |  |  |  | **VEGFA** | NM_001025366 | down |
| **RAC2** | NM_002872 | down |  |  |  | **VHL** | NM_000551 | down |
| **RALB** | NM_002881 | down |  |  |  | **ACVR1C** | NM_145259 | up |
| **RASSF1** | NM_170713 | down |  |  |  | **APC2** | NM_005883 | up |
| **RASSF5** | NM_182663 | down |  |  |  | **BIRC3** | NM_001165 | up |
| **RB1** | NM_000321 | down |  |  |  | **CCNA1** | NM_003914 | up |
| **RXRA** | AK090416 | down |  |  |  | **CDKN2A** | NM_058197 | up |
| **RXRB** | NM_021976 | down |  |  |  | **CEBPA** | NM_004364 | up |
| **STK4** | BC005231 | down |  |  |  | **EGF** | NM_001963 | up |
| **SUFU** | NM_016169 | down |  |  |  | **FGF12** | NM_004113 | up |
| **TGFBR3** | NM_003243 | down |  |  |  | **FGF13** | NM_004114 | up |
| **TPM3** | NM_152263 | down |  |  |  | **FGF14** | NM_175929 | up |
| **WNT1** | NM_005430 | down |  |  |  | **FGFR3** | NM_000142 | up |
| **ACVR1C** | NM_145259 | up |  |  |  | **FOS** | NM_005252 | up |
| **APC2** | NM_005883 | up |  |  |  | **FZD7** | NM_003507 | up |
| **AXIN2** | NM_004655 | up |  |  |  | **FZD9** | NM_003508 | up |
| **CCNA1** | NM_003914 | up |  |  |  | **IGF1R** | NM_000875 | up |
| **CDC42** | NM_044472 | up |  |  |  | **IL8** | NM_000584 | up |
| **CEBPA** | NM_004364 | up |  |  |  | **JUN** | NM_002228 | up |
| **COL4A6** | NM_033641 | up |  |  |  | **JUP** | NM_002230 | up |
| **CTNNA3** | NM_013266 | up |  |  |  | **KLK3** | AF335478 | up |
| **EGLN3** | NM_022073 | up |  |  |  | **LAMA2** | NM_000426 | up |
| **EPAS1** | NM_001430 | up |  |  |  | **LAMA3** | NM_198129 | up |
| **ERBB2** | NM_001005862 | up |  |  |  | **MITF** | NM_198159 | up |
| **FAS** | NM_000043 | up |  |  |  | **PRKCA** | NM_002737 | up |
| **FGF13** | NM_004114 | up |  |  |  | **RAC2** | NM_002872 | up |
| **FGFR3** | NM_000142 | up |  |  |  | **SMAD2** | NM_001003652 | up |
| **FN1** | NM_212482 | up |  |  |  | **SMAD3** | NM_005902 | up |
| **FZD3** | NM_017412 | up |  |  |  | **TGFB2** | NM_001135599 | up |
| **GLI2** | NM_005270 | up |  |  |  | **TRAF1** | NM_005658 | up |
| **HGF** | NM_001010931 | up |  |  |  | **WNT11** | NM_004626 | up |
| **JAK1** | NM_002227 | up |  |  |  | **WNT4** | NG_008974 | up |
| **KIT** | NM_000222 | up |  |  |  | **WNT6** | NM_006522 | up |
| **LAMA1** | NM_005559 | up |  |  |  |  |  |  |
| **LAMB4** | NM_007356 | up |  |  |  |  |  |  |
| **LEF1** | NM_016269 | up |  |  |  |  |  |  |
| **MECOM** | NM_005241 | up |  |  |  |  |  |  |
| **MITF** | NM_198159 | up |  |  |  |  |  |  |
| **MMP1** | NM_002421 | up |  |  |  |  |  |  |
| **PDGFRA** | AA599881 | up |  |  |  |  |  |  |
| **PIK3CG** | NM_002649 | up |  |  |  |  |  |  |
| **PLCG2** | NM_002661 | up |  |  |  |  |  |  |
| **RARB** | NM_000965 | up |  |  |  |  |  |  |
| **RUNX1T1** | AF018283 | up |  |  |  |  |  |  |
| **SMO** | NM_005631 | up |  |  |  |  |  |  |
| **TCF7** | NM_003202 | up |  |  |  |  |  |  |
| **TGFA** | NM_003236 | up |  |  |  |  |  |  |
| **WNT11** | NM_004626 | up |  |  |  |  |  |  |
| **WNT6** | NM_006522 | up |  |  |  |  |  |  |
| **WNT9A** | AB060283 | up |  |  |  |  |  |  |

**Table S2**
