# Supplementary material for: miR-126&126* Restored Expressions Play a Tumor Suppressor Role by Directly Regulating ADAM9 and MMP7 in Melanoma
Source: PLoS One. 2013 Feb 21;8(2):e56824. doi: 10.1371/journal.pone.0056824 (PMC3578857; doi:10.1371/journal.pone.0056824)
Supplement: Table S3 — Melanogenesis. Results of Me665/1 melanoma cell line transduced with miR-126&126* compared with controls. This list of differentially expressed genes derives from KEGG “Melanogenesis”. The differential gene expression is obtained as log2 of the ratio between miR-126&126* and control cells. Up- and down-regulated genes correspond to >2 and < 2, respectively. (DOCX) [file pone.0056824.s007.docx]

| **Gene Symbol** | **Genbank Accession** | **Me665/1**  **miR-126&126* vs Tween** |
| --- | --- | --- |
| **EP300** | NM_001429 | down |
| **DVL3** | NM_004423 | down |
| **EDN1** | NM_001955 | down |
| **EDNRB** | NM_003991 | down |
| **FZD1** | NM_003505 | down |
| **FZD5** | NM_003468 | down |
| **GNAI2** | NM_002070 | down |
| **GNAQ** | NM_002072 | down |
| **MAPK3** | NM_002746 | down |
| **HRAS** | NM_005343 | down |
| **WNT1** | NM_005430 | down |
| **GNAS** | AJ224867 | up |
| **ADCY9** | NM_001116 | up |
| **CAMK2B** | NM_172082 | up |
| **CAMK2D** | NM_001221 | up |
| **DCT** | NM_001922 | up |
| **FZD3** | NM_017412 | up |
| **LEF1** | NM_016269 | up |
| **MITF** | NM_198159 | up |
| **PLCB1** | NM_015192 | up |
| **PLCB2** | NM_004573 | up |
| **KIT** | NM_000222 | up |
| **TCF7** | NM_003202 | up |
| **MC1R** | NM_002386 | up |
| **TYRP1** | NM_000550 | up |
| **WNT11** | NM_004626 | up |
| **WNT6** | NM_006522 | up |
| **WNT9A** | AB060283 | up |

**Table S3**
