# Supplementary material for: miR-126&126* Restored Expressions Play a Tumor Suppressor Role by Directly Regulating ADAM9 and MMP7 in Melanoma
Source: PLoS One. 2013 Feb 21;8(2):e56824. doi: 10.1371/journal.pone.0056824 (PMC3578857; doi:10.1371/journal.pone.0056824)
Supplement: Table S4 — Primer list. This list includes all the utilized primers for miR-126&126* and 3′UTRs cloning and for semiquantitative RT-PCR. (DOCX) [file pone.0056824.s008.docx]

**Primers for miR-126&126* cloning**

|  | **Primer sequence** |
| --- | --- |
| **Dir** | 5’-ACATTGCCGTGTGGCTGTTA-3’ |
| **Rev** | 5’-TCTGCACTTCTTCCTTCATT-3’ |

**Cloning primers for 3’UTRs:**

| **GeneSymbol** | **Primer sequence** | **References** |
| --- | --- | --- |
| **ADAM9 Dir** | 5'-TCACGCAGTTACTCGCTTCC-3' |  |
| **ADAM9 Rev** | 5'-AAGCTACTAGGAGACACAAAAG-3' |  |
| **MMP7 Dir** | 5'-ATGGAAAGAGAAGTAATTCAAGA-3' |  |
| **MMP7 Rev** | 5'-ATTGACATCTACCCACTG-3' |  |
| **OPN Dir** | 5'-GTGCATCTTCTGAGGTCAATT-3' |  |
| **OPN Rev** | 5'-ATTTGCTGGACAACCGTGGGA-3' |  |
| **PI3KR2 Dir** | 5'-CTGATGCAGATTCAGGGCTT -3' |  |
| **PI3KR2 Rev** | 5'-GATGTTTTATATCATATCAA-3' |  |

**Primers for semiquantitative RT-PCR**

| **GeneSymbol** | **Primer sequence** |
| --- | --- |
| **OPN Dir** | 5-GTGCATCTTCTGAGGTCAATT-3' |
| **OPN Rev** | 5’-ATTTGCTGGACAACCGTGGGA-3’ |
| **ADAM9 Long Dir** | 5’-TCCAGTGACACCTCCCAGAGAA-3’ |
| **ADAM9 Long Rev** | 5’-GGTTGCTTGGCTGCATAGGTT -3’ |
| **ADAM9 Short Dir** | 5’-CTGAGGCAGAGCTGTTTGTACCT-3’ |
| **ADAM9 Short Rev** | 5’-ATGTTTAGGCAAAATTCCAGGG-3’ |
| **GAPDH Dir** | 5’-acatcaagaaggtggtgaagcagg-3’ |
| **GAPDH Rev** | 5’-ctcttcctcttgtgctcttgctgg-3’ |

**Table S4**
